# Sharing Milk and Knowledge in the Neonatal Intensive Care Unit Improves Care for Neonates in a Low- and Middle-Income Population—A North–South Collaboration

**DOI:** 10.3390/children12030326

**Published:** 2025-03-04

**Authors:** Kirsti Haaland, Srishti Goel, Gunjana Kumar, Ingvild Andresen Hurv, Isha Thapar, Jitesh Jalthuria, Sushma Nangia

**Affiliations:** 1 Department of Global Health, Division of Emergencies and Critical Care, Oslo University Hospital, Ullevål, Kirkeveien 166, 0450 Oslo, Norway; 2 Department of Neonatal Intensive Care, Division of Paediatric and Adolescent Medicine, Oslo University Hospital, Ullevål, Kirkeveien 166, 0450 Oslo, Norway; 3 Lifeline Advanced Neonatal Centre, 63 & 64, Cool Road, Waryam Nagar, Jalandhar 144001, Punjab, India; srishti87@gmail.com; 4 National Institute of Medical Science & Research, NH-11C, Delhi-Jaipur Expressway, Jaipur 303121, Rajasthan, India; gunjanakumar@gmail.com; 5 Institute for Nursing, Faculty of Health, VID Specialised University, Theodor Dahls vei 10, 0370 Oslo, Norway; 6 National Comprehensive Lactation Management Centre, Lady Hardinge Medical College, Shaheed Bhagat Singh Marg, Connaught Place, New Delhi 110001, India; drishaathapar@gmail.com (I.T.); jiteshjaluthria@gmail.com (J.J.); 7 Department of Neonatology, Lady Hardinge Medical College, Shaheed Bhagat Singh Marg, Connaught Place, New Delhi 110001, India; drsnangia@gmail.com

**Keywords:** preterm infant, neonate, mortality, morbidity, human milk, KMC, educational exchange, nursing, collaboration, breastfeeding, donor breast milk, global health, clinical health, nutrition, infection control, QI

## Abstract

**Background:** Basic healthcare may significantly decrease neonatal morbidity and mortality. Attention to this, particularly in populations where rates of potentially preventable illness and death within the first weeks of life are extremely high, will have a positive impact on global health. **Objective:** This manuscript presents the development and impact of a quality improvement programme to reduce the evidence–practice gap in care for neonates admitted to the NICU in a public hospital in India. The programme was locally customised for optimal and sustainable results. **Method:** The backbone of the project was educational exchange of neonatal nurses and physicians between Norway and India. Areas of potential improvement in the care for the neonates were mainly identified by the clinicians and focus areas were subject to dynamic changes over time. In addition, a service centre for lactation counselling and milk banking was established. Progress over the timeframe 2017–2019 was compared with baseline data. **Results:** The project has shown that after a collaborative effort, there is a significant reduction in mortality from 11% in the year 2016 to 5.5% in the year 2019. The morbidity was reduced, as illustrated by the decrease in the proportion of neonates with culture-proven sepsis. Nutrition improved with consumption of human milk by the NICU-admitted neonates remarkably increasing from one third to more than three forth of their total intake, and weight gain in a subgroup was shown to increase. With the introduction of family participatory care, hours of skin-to-skin contact for the neonates significantly increased. Additional indicators of improved care were also observed. **Conclusions:** It is feasible to reduce neonatal mortality and morbidity in a low- and middle-income hospitalised population by improving basic care including nutrition relatively inexpensively when utilising human resources.

## 1. Introduction

In 2019, 2.4 million children died in the neonatal period defined as the first 28 days of life [1]. The global neonatal mortality rate (NMR) was 17 deaths per 1000 live births that year. Corresponding numbers for India and Norway were 21.7 and 1.4, respectively [1]. Although NMRs have declined significantly since 1990, increased efforts are necessary to achieve the third Sustainable Development Goal “Good health and well-being” [2]. In many neonatal care units in low- and middle-income countries, basic care practice is suboptimal and a main underlying reason for acute and chronic morbidity and mortality. Interventions such as promoting early enteral feeding and intake of mother’s own milk (MOM), maintaining hand hygiene and both providing necessary and minimising irrational use of antibiotics, involvement of parents, and reducing the exposure to stress have been depicted to improve various neonatal outcome parameters [3].

**Figure 1:**
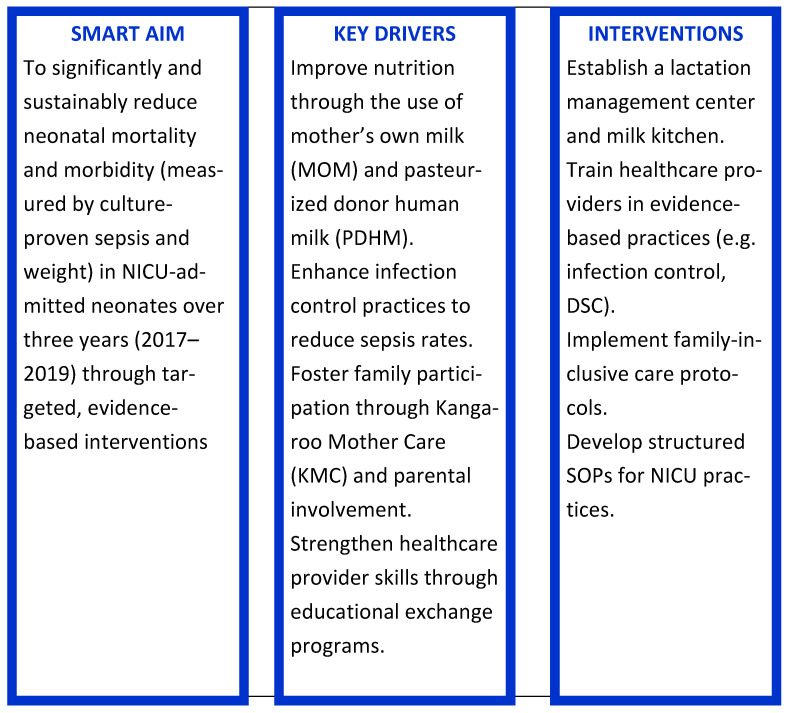
A Quality Improvement Initiative [5]. SMART Specific Measurable Achievable Relevant Time-bound.

The project entitled “Oslo-Delhi: Improve newborn care” [4] aimed to narrow the gap between theoretical knowledge and active practice of optimised neonatal basic care through collaboration across cultural and social contexts. The two key components were educational exchange of nurses and physicians between neonatal intensive care units (NICU) in New Delhi, India and Oslo, Norway, and establishing a lactation centre with a bank for human milk. The main goal of this Quality Improvement Initiative was to enhance the basic care of sick and premature neonates (Figure 1).

## 2. Methods

Target population and training setting: All neonates admitted to the 80 bedded, level III NICUs at Lady Hardinge Medical College and Kalawati Saran Children’s Hospital (LHMC and KSCH), New Delhi, India during the timeframe of January 2017 to December 2019. The 47 bedded level IV NICU at Oslo University Hospital, Oslo, Norway was the location for exchange. Neonates admitted to this hospital are not reported in this manuscript.

The framework for the project was constructed on a template provided by the Norwegian Agency for Exchange Cooperation, employing educational exchange for learning and development [6]. A total of 12 Indian nurses, 6 Indian physicians, and 9 Norwegian nurses spent 3–18 months at the foreign location in a 3-year relay. In addition, Norwegian resource personnel skilled in human milk banking, administration, education, neonatal care, and infection control participated as per the needs during various timeframes of the project.

Partners’ discussion and previous experience derived from a similar project involving Oslo University Hospital and JK Lone Hospital, Jaipur, India [7], identified the areas of potential improvements. This determined the objectives of the project. Focus areas and cost-effective ways to achieve quality improvements were sought by applying a patient-centred model of care, rather than the more traditional approach merely supporting the provision of services in keeping with established guidelines. “Participant observation” was the most important tool in facilitating collaboration for the continuous development and customisation of this dynamic project.

Empowerment and skills of health providers were enhanced by exposure to a wide range of patient scenarios and different settings to be handled in collaboration with foreign colleagues, and by theory sessions. The framework of the project was reviewed every year.

### 2.1. Education and Training

Areas of focus included nutrition, hygiene, pain management, kangaroo mother care (KMC), developmental supportive care (DSC), non-invasive mechanical ventilation, resuscitation, temperature control, teamwork, and maintenance of medical equipment (Supplement S1).

Collaborative task- and discussion-based learning were the main approaches. Indian and Norwegian health providers worked together bedside in the two countries’ NICUs, customising activities according to their individual strengths and skills. Patient management as well as different systems and infrastructure at the respective units were observed and discussed.

Teaching sessions and scenario trainings in small groups were frequently arranged, with “topic of the week” (Table 1). Most topics were repeated several weeks. Protocols and standard operational procedures customised to local conditions were constructed (Table 1).

During the tenure of 3 years, survey questions were answered twice by the participants. This provided information on their knowledge of different topics and the activities were modified accordingly (Supplement S2).

With the purpose to empower healthcare providers to continuously take appropriate actions on crucial improvement areas and to facilitate for sustainability, three inter-disciplinary study groups were established, with a primary focus on nutrition, hygiene and housekeeping, and DSC. They met approximately twice a month.

Seminars were conducted twice a year to educate the healthcare providers and equally important to enhance their teaching skills necessary for long term improvements. The last year this was expanded to a two-day seminar with participants and speakers also from outside the project [8].

### 2.2. Providing Human Milk

A comprehensive lactation management centre (CLMC), ‘Vatsalya Maatri Amrit Kosh’ (literal meaning: ‘a storehouse of mother’s unconditional love and nectar’), was established in the hospital to provide lactation support and to provide pasteurised donor human milk (PDHM) in cases where sufficient MOM is not available. To accomplish this, lactation counsellors and milk bank operators were engaged and trained, and facilities for collection of surplus milk from suitable mothers, processing, storage and dispersion PDHM were established [9] (Table 2).

Lactation counselling was offered to groups of women in all antenatal, maternity, neonatal, and some paediatric wards, with complementary individual sessions as and when required. Awareness of the benefits of human milk and (preferably exclusive) breastfeeding, practical assistance and ally of the women’s anxieties were addressed. Mothers of neonates not yet able to suckle directly on the breast due to prematurity or severe illness, were encouraged and helped to express their milk manually or via a breast pump to initiate and sustain production until their neonate managed to suckle. This expressed milk was fed to the infant by gavage or cup.

Women with surplus production were invited to donate to the milk bank. Neonates of GA < 32 weeks or weight < 1500 g were offered (if informed consent from the parents) PDHM as a bridge to support during the first two weeks of life, if MOM was insufficient.

Milk kitchens were set up in the neonatal units to organise thawing and distribution (and in some cases fortification) of PDHM and artificial formula in an aseptic manner.

### 2.3. Outcome Measures

Mortality rates among neonates admitted to the NICU were documented on monthly basis. Clinical as well as culture-proven sepsis and antibiotic utilisation rates (defined as the proportion of neonates that had received at least one kind of antibiotics to the total number of neonates discharged) were recorded on a daily basis. As quality indicators of neonatal care, nasal injuries due to non-invasive respiratory support and duration of KMC were documented by the nursing personnel in the bedside monitoring sheets. Quality of nutrition was assessed based on daily consumption of MOM, PDHM, and formula, respectively, in the ward, and by the weight change in the first two weeks of life recorded early in the project (2nd month) and repeated 2.5 years later.

Involved personnel reported their own activities and observations of the project’s impact every third month. These reports were utilised for unstructured and qualitative measures such as attitude towards changes, parental involvement, and effect of training (e.g., resuscitation and DSC).

Altered design and practice of physical and work environment such as logistics, teamwork, maintenance of equipment, and more, were noted.

The functionality of the human milk bank was followed through data on the amount of milk donated and that dispensed to the neonatal unit.

Statistical tools: *p*-values calculated using Pearson’s Chi Square for categorical variables, ANOVA for parametric references.

## 3. Results

### 3.1. Mortality, Morbidity and Demographics

**Figure 2:**
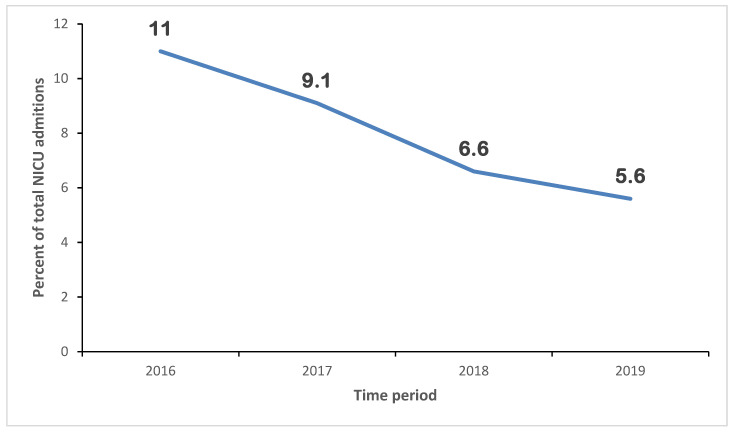
Neonatal mortality rates over 4 years (2016–2019).

The mortality rate in the NICU was reduced to almost half across the study period from 2016 to 2019 (Figure 2). Notably, despite an increase in the number of neonates admitted to the intensive care unit, a larger proportion being small for gestational age (SGA) and more neonates needing short respiratory support after birth. There is an improvement in the proportion of women receiving appropriate antenatal care and those offered with complete coverage of antenatal steroids. Other demographic characteristics did not differ significantly (Table 3).

**Figure 3:**
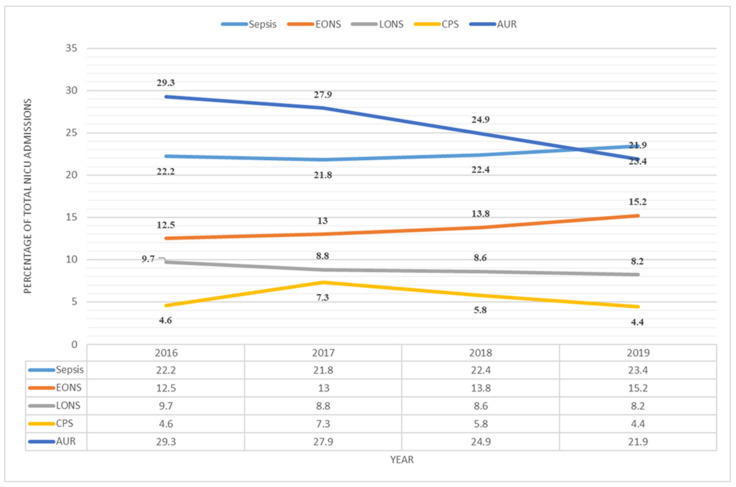
Sepsis in NICU-admitted neonates. Sepsis’ includes all cases with clinical, probable, or culture-proven sepsis. Clinical sepsis is defined as presence of signs and/or symptoms attributable to neonatal sepsis with negative sepsis screen and blood culture. Probable sepsis is defined as presence of signs and/or symptoms attributable to neonatal sepsis along with a positive sepsis screen, but blood culture is negative. CPS: Culture proven sepsis is defined as isolation of microorganism from blood culture in a baby with signs and/or symptoms attributable to sepsis with or without a positive sepsis screen. EONS: Early onset sepsis refers to presentation before 72 h of life. LONS: Late onset sepsis refers to presentation after 72 h of life. AUR: Antibiotic utilisation rate defines the proportion of neonates that received at least one antibiotic therapy to the total number of patients discharged.

Morbidity, as indicated by the proportion of neonates with culture-proven sepsis, as well as overall antibiotic utilisation rates in NICU, showed a statistically significant decline from 2016 to 2019 (Figure 3).

### 3.2. Nutrition

**Figure 4:**
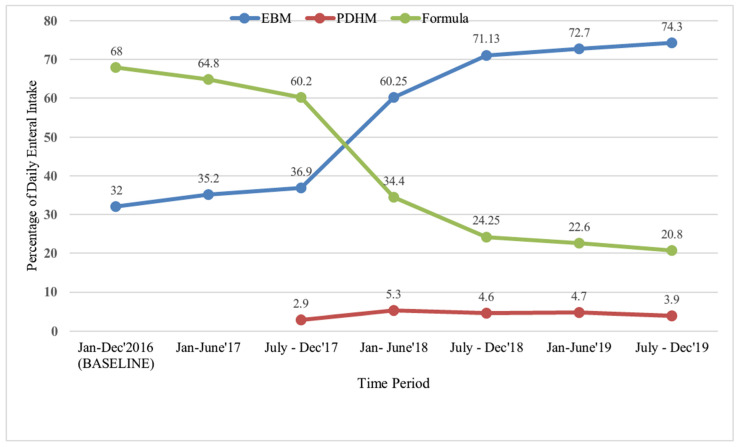
Enteral nutrition of neonates in the NICU. Percentage of different types of milk consumed in the NICU in different time intervals. EBM, expressed breast milk; PDHM, pasteurised donor human milk.

Daily consumption of MOM by the infants in the NICU increased significantly while the use of baby formula decreased (Figure 4). Sick and small neonates were prioritised to receive available PDHM if their MOM volume was insufficient.

**Figure 5:**
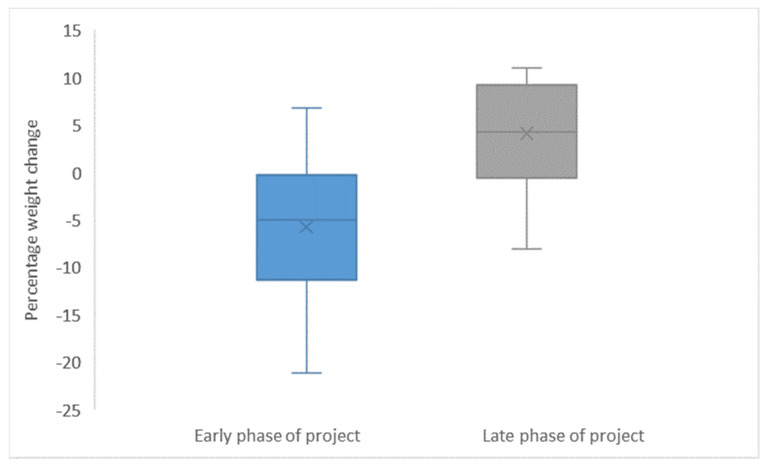
Weight change in the first 14 d of life in infants GA < 32 w early versus late in the project period. Mean and SD weight change for individual neonates born < 32 w GA investigated in the early phase (2nd month) and in the late phase (2 ½ year later).

The overall percentage change in weight of GA infants < 32 w from birth to two weeks decreased by mean (SD) 5.7 (7.0) in the initial phase of the project period and increased by mean (SD) 4.1 (5.2) in the late phase (Figure 5).

### 3.3. Additional Indicators of Care

DSC and “Structured observation sheets” were implemented for better observation, understanding of the needs and optimal handling of neonates according to their behaviour. As their daily routines, nurses also ensured control of external stimuli, clustering of nursing care activities, non-pharmacological pain management, and strengthening of parent involvement. Parents became active in the care of their hospitalised neonates. Improved understanding, along with a change in attitude towards KMC evolved. Other qualitative observations include improved resuscitation after focusing on standard operational procedures and teamwork in scenario training. Table 4 depicts quantitative data on KMC and nasal injury. Duration of skin-to-skin contact for individuals increased. A greater proportion of neonates, including those extremely premature and those on non-invasive respiratory support, received KMC. More caretakers, mothers, and others, engaged. There was a significant reduction in the proportion of nasal injuries due to non-invasive respiratory support, particularly the most severe (Table 4).

### 3.4. Implemented Routines

Routines for infection control were attended to in several ways. Focus on hand hygiene intensified and accomplished by adding new washing facilities at the entrances and making hand disinfectants more available. Immediate sorting of waste and laundry in appropriate bags in stands replaced heaps on the floor. Construction work was performed to hinder vermin in the wards. Protocols for routines and responsibility for regular cleaning of incubators, milk kitchen equipment, and more were framed and implemented.

According to the qualitative reports, better teamwork was obtained, as illustrated by the more consistent and accurate handover reports, dividing responsibility for the particular patients, and empowerment of colleagues through competence sharing and debriefing.

Efforts to maintain medical equipment were made by designating responsibilities to individual personnel to follow up routines for use, service, and repair (such as ensuring sufficient water in the cPAP-machine, choosing crucial alarms, organising service of ventilators).

### 3.5. The Comprehensive Lactation Management Centre (CLMC)

The CLMC offers individual assistance and helps all lactating women on a daily basis irrespective of whether their baby was admitted to the NICU or not. The most common challenges that interfere with the early initiation of breastfeeding include primiparous mothers, preterm born neonates admitted to the NICU, and engorgement of breasts.

**Figure 6:**
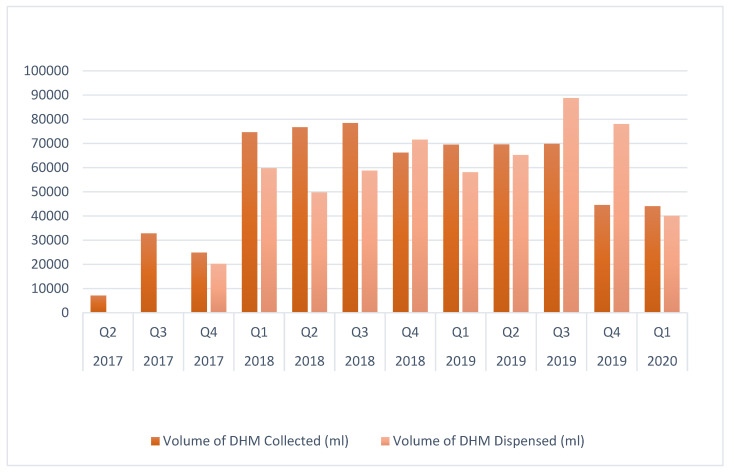
Quarterly depiction of the volume of donor human milk (DHM) collected and disbursed.

The main goal was to provide MOM to all infants, using PDHM mainly as a bridge. The turnover in the milk bank was quite stable (Figure 6).

Contribution to “National guidelines and SOPs of Indian milk banks” was a ripple effect of developing the lactation centre, as SOPs developed locally to some extent served as references for drafting the national ones [10].

## 4. Discussion

The goal of the project “Oslo-Delhi: Improve newborn care” was to enhance the basic care of sick and premature neonates by narrowing the gap between the theory of optimal neonatal care and locally customised practise, and by establishing a lactation centre. Basic care is required for the wellbeing of all neonates, necessary for the survival of sick and premature neonates, and fundamental for all advanced medical treatment of neonates. A bundle of awareness, knowledge, skills, practical actions and efforts was implemented. The impact of the project is illustrated with various indicators. Due to the synergistic nature of the interventions, separate evaluation of the variables cannot be defined.

The mortality rate in the NICU decreased despite a larger and more vulnerable population. The proportion of SGA neonates and neonates suffering from early onset neonatal sepsis (EONS) was higher. EONS is mainly caused by factors occurring before admittance to the NICU; hence, it is not controllable [11]. The share of neonates with late onset neonatal sepsis (LONS) and culture-proven sepsis was reduced; however, and lower antibiotic utilisation rates resulted in reduced morbidity. The focus on infection prevention and augmenting the developing immune system (i.e., by providing human milk and limiting exposure to antibiotics), enhances this. Antimicrobial stewardship indicates better care as these drugs may also harm and, therefore, should be subjected to strict indications [12,13,14]. Decreased mortality and morbidity indicate better care, as do less cases of nasal injuries (for which nursing skills are crucial), more KMC, and better weight gain. The documented increase in nutrition with human milk is tantamount to better nutrition in this population [15]. In addition to increased growth, adequate nutrition is necessary for achieving optimal neurodevelopment [16] and greatly enhances the immune system [17,18]. Our results are in accordance with WHO: “child deaths can be prevented by providing immediate breastfeeding, improving access to skilled health professionals for antenatal, birth, and postnatal care, improving access to nutrition and micronutrients, promoting knowledge of dangers”.

Factors contributing to optimal care for neonates are described in the literature. The amount of information appears overwhelming, and it is challenging to prioritise and combine elements that are effective and feasible for a particular location. It may be more time consuming than what can be afforded to determine appropriate improvements. An external collaborating partner may observe the situation from a different angle and base considerations on alternate experiences, thus contributing to different solutions. Together, collaborators obtain a broader perspective of the present situation and a larger base of experience to separate futile and potentially significant measures to develop plans [19]. Returned exchanged personnel add to this as they have been exposed to different solutions, practised unfamiliar routines, and experienced results of these.

The exchange of health providers is beneficial for more reasons. Implementing changes in basic routines, especially those requiring more effort without necessarily immediately having a big impact, calls for patience, trust, and understanding amongst the staff which may require raised health literacy as well as altered attitude. It is recognised that not only knowledge and skills but also an attitude to own labour efforts and self-confidence are crucial [20,21]. Performing one’s professional skills in a different context and milieu often results in new perceptions of one’s own labour, which may influence attitude. Accordingly, personnel engaged in the project reported a positive attitude towards change and higher empowerment as a major change.

In addition to educational exchange, the project comprised development of a lactation centre. Nutrition is a ladder to higher levels of health support. The lactation centre facilitates many steps on this ladder. It serves as a source for developing and sharing knowledge and skills between colleagues and mothers. Furthermore, donated surplus milk serves a dual purpose. The receiving neonates avoid early exposure to formula and the donors keep up their production till their own neonates need more milk. Potential donors would otherwise not express milk, thereby staggering their production in a critical phase as well as increasing the risk of engorged breasts or discarding the milk because they lack access to a private freezer for storage. After MOM, PDHM is the second-best nutrition. Milk kitchens in the neonatal units are extended arms of the milk bank for safe defrosting and distribution of PDHM to the neonates. The development of a National guidelines and SOPs of Indian milk banks illustrates how a system and infrastructure alterations the results from individual efforts and attitudes.

Leading causes of neonatal morbidity and mortality, such as prematurity and infections, are largely preventable. India harbours health centres delivering top world-class perinatal care. The variation between locations causes unnecessary high national NMR. We believe that projects like the one described here, demonstrating improvements achieved by relatively limited economic resources, may be a step in the direction of the grander vision “Good health and wellbeing for all (SDG 3)”. Providing evidence for cost-effective measures may be a way for the global society to incentivize, urge, and support national efforts for extended sustainable overall improvement.

## 5. Conclusions

Care of hospitalised neonates was enhanced through educational exchange and facilitation for exploiting the potential of human milk. Mortality and morbidity were reduced. Reciprocal partnership, prioritising competence sharing, and comprehensive focus areas may effectively support “Better health for all”.

## Figures and Tables

**Table 1:** “Topic of the week”, new protocols and Standard Operational Procedure during the three-year project period.

“Topic of the Week”	Associated Protocols
Parental involvement in the care of the sick- and premature infant	DSC
Parental involvement through kangaroo mother care (KMC), also for neonates on respiratory support	KMC
Nesting and positioning of the premature infant	DSC
Reducing noise in the NICUs	
Maintenance of thermo-neutral zone	Incubator KMC
Documentation of Clinical observations including pain score	Structured observation sheets
A (airways), B (breathing), C (circulation),	
Introduction of NIDCAP	
Incubator care of premature infants	Use of incubator and weighing of neonates inside incubator
Cleaning of incubator	Housekeeping protocol for cleaning of incubator
Use of aseptic technique when preparing Intravenous fluid and parenteral nutrition	
Housekeeping and general hygiene	
Hand hygiene	
Pain assessment	
Pain management	Swaddling KMC. Dextrose
Initiating feeding of premature infants	Non-nutritive sucking Colostrum harvesting
The link between KMC and breastfeeding	KMC Breastfeeding
Operationalizing of milk kitchen	Milk kitchen protocols
Care of infants on cPAP	cPAP Documenting cPAP injury in the monitoring sheets
Shift change; structured handover and safe organisation of units	Neonates’ monitoring sheets

DSC, developmental supportive care; KMC, kangaroo mother care; NIDCAP, newborn individualised developmental care and assessment programme.

**Table 2:** The “lactation centre curriculum”.

Topic	Knowledge/Skills Expected from the Lactation Counsellors
Components of human breast milk	Macro- and micro-nutrients Other components
Human breastmilk and baby formula	Advantages of human breast milk over baby formula.
Composition of human breast milk in the first 6 months	Expected changes between and during meals and over the first days and moths. Individual variation and differences in premature mothers’ and term mothers’ milk.
Colostrum	Harvesting Unique benefits, i.e., for digestion and immune system development.
Preparation for early breast feeding	Antenatal care for human breast milk expression. Benefits associated with stimulation for expression of milk the first hour after birth
Milk volume	Expected volumes the first days and weeks depending on factors such as gestational age, parity and delivery mode. Neonatal ventricle size
Importance of mothers’ own milk	Benefits of mothers’ own milk especially for sick or premature neonates.
Storage of expressed human breast milk	Contamination risks, safe collection and the limit of 4 h safe storage at room temperature.
Fortification of milk for preterm and low weight infants	Different nutrient requirements for different groups.
Pasteurised human milk	Importance and safety
Supplemental feeds	Very rarely required the first 6 months Benefits of long-term breastfeeding (>6 months)
Non-nutritional advantage of breastfeeding	For infant, mother and society. Short- and long-term. Including financial benefits.
Mastitis and differential diagnoses	Identification of the issues and insight in the treatment options
Painful feeding	Comprehension and identification of easily correctable issues like latching and positioning
Providing lactation management and counselling	Practical skills
Signs mothers need to be aware of	Early feeding signals like rooting and hand to the mouth. Warning sign like lethargy, dullness, abnormal body movements, inappropriate suck–swallow–breathing coordination. Inadequate temperature.
	
**Topic**	**Knowledge/Skills Expected from Milk Bank Operators**
Donors	Lactating mothers who voluntarily donate their surplus breast milk after undergoing a thorough screening process, which includes medical history evaluation and serological testing.
Handling and storage protocol–Step 1	Freezing at −20 °C to preserve its nutritional and immunological properties until further processing
Handling and storage protocol–Step 2	Pasteurisation using the Holder’s method (62.5 °C for 30 min followed by rapid cooling), to eliminate potential pathogens while preserving essential bioactive components
Handling and storage protocol –Step 3	Testing milk for microbial contamination before dispension

**Table 3:** Year-wise demographic profile of infants admitted to the neonatal unit (2016–2019).

	Baseline (January–December 2016)	Year 1 (January–December 2017)	Year (January–December 2018)	Year 3 (January–December 2019)	*p*-Value
Infants admitted, n (Percent of live births)	2420 (17.7)	2550 (19.5)	2893 (21.5)	3131 (24.9)	<0.0001
GA *	34.3 (3.62)	34.5 (3.7)	34.5 (4.1)	34.4 (3.8)	0.482
GA < 32 weeks (%)	589 (24.3)	607 (23.8)	673 (23.29)	732 (23.3)	0.793
GA < 28 weeks (%)	102 (4.2)	113 (4.4)	120 (4.1)	134 (4.3)	0.961
Birth Weight **	1987 (702)	2006 (726)	1980 (709)	1995 (713)	0.365
VLBW (%)	642 (26.5)	659 (25.8)	774 (26.8)	778 (24.8)	0.335
ELBW (%)	144 (5.9)	138 (5.4)	162 (5.6)	167 (5.3)	0.778
SGA (%)	803 (33.2)	940 (36.9)	1104 (38.2)	1240 (39.6)	<0.001
Caesarean (%)	1073 (44)	1170 (46)	1295 (45)	1348 (43)	0.194
Antenatal Steroids ***	40	43.2	46.9	48.3	<0.001
Antenatal care ****	38.2	39.1	38.3	42.8	0.0006
Resuscitation (%)	523 (21.6)	596 (23.4)	704 (24.3)	799 (25.5)	0.007

* Gestational age in mean weeks (SD), ** Mean grams (SD), *** Percentage of neonates ≤ 34 weeks admitted to the NICU fully covered (meaning four doses of injection dexamethasone 6 mg, given IM, 12 h apart or, two doses of injection betamethasone 12 mg, given IM, 24 h apart, at least 24 h prior to delivery), **** Percentage received optimum antenatal care (a minimum of four antenatal visits, two doses of tetanus toxoid and 100 tablets of iron and folic acid during pregnancy). VLBW, very low birth weight (<1500 g); ELBW, extremely low birth weight (<1000 g); SGA, birth weight <10th Centile, resuscitation defined as need for initial steps and beyond in neonates who do not establish spontaneous breathing at birth.

**Table 4:** Frequency of nasal injuries and duration of KMC.

	Baseline (January–December 2016) n = 2420	Year 1 (January–December 2017) n = 2550	Year 2 (January–December 2018) n = 2893	Year 3 (January–December 2019) n = 3131	*p*-Value
Nasal injuries, all grades * n (%)	99 (67)	85 (56.3)	67 (38.9)	43 (25.7)	<0.00001
Nasal injuries, grade 3 * n (%)	26 (17.7)	19 (12.6)	14 (8.1)	6 (3.6)	0.00032
KMC duration Mean (SD) **	2.89 (1.89)	4.67 (2.12)	6.23 (2.43)	7.67 (1.24)	<0.00001

n, number of neonates admitted to the NICU in the separate years; KMC, kangaroo mother care, * Pressure sores infants of GA < 32 weeks requiring respiratory support graded from 1: skin discolouration to 3: necrosis or damage to the skin patch and underlying bone. ** Average duration of KMC hours per day per eligible neonate during the hospital stay.

## Data Availability

All material is available from the authors on reasonable request. The data are not publicly available due to technical limitations.

## Supplementary Materials

The following supporting information can be downloaded at: https://www.mdpi.com/article/10.3390/children12030326/s1, Supplement S1: Expected project; Supplement S2: Survey.
