# Supplementary material for: Sharing Milk and Knowledge in the Neonatal Intensive Care Unit Improves Care for Neonates in a Low- and Middle-Income Population—A North–South Collaboration
Source: Children (Basel). 2025 Mar 4;12(3):326. doi: 10.3390/children12030326 (PMC11940916; doi:10.3390/children12030326)
Supplement: Supplementary file 1 [file children-12-00326-s001.zip › Froof S1.pdf]

- Improved overall health and wellbeing of the neonates admitted to the NICU

1. National human milk bank and milk kitchen are established and are operationalized as per National guidelines
2. All babies admitted to NICU that can be fed enterally aimed to receive exclusive human milk.
3. More babies at discharge from NICU are being predominantly breastfed.
4. All mothers in the maternity wards exclusively breastfeed their own healthy babies before discharge home.
5. Improved housekeeping & hygiene according to unit protocol
6. Non-pharmacologic & pharmacologic measures for pain management to be implemented as unit protocol.
7. Increased use of kangaroo mother care
8. Improved care of neonates on respiratory support
9. Ameliorated care of neonates at the time of resuscitation
10. Normothermia in inborn neonates while being transferred to NICU
11. Developmental Supportive Care implemented for neonates admitted to the NICU
12. Improved teamwork in the NICUs
13. Operating and maintenance of medical-technical equipment's in the unit according to unit protocol

Supplement S1. Expected project results

DO NOT WRITE YOUR NAME

IN SOME OF THE QUESTIONS YOU MAY CHOOSE MORE THAN ONE OF THE OPTIONS

What is your profession?

How long have you worked here?

Which ward do you work at?

When can a baby start to suckle at the breast?

How long do you recommend the mothers to exclusively breast feed their babies?

What is an expected weight loss for a newborn after birth?

What is the best nutrition for preterm babies?

What is Kangaroo mother care?

KMC is not recommended in which all neonates?

What do you do to support and help the patients, apart from ventilation, medication, feeding and sponging?

What positions is best for the baby?

How often do you change the baby's position?

Which of the following aspects are influenced by a calm environment and careful handling?

Weight gain, length of hospital stay, neurodevelopmental scores, risk of cerebral hemorrhage, bonding, icterus, digestion

What do you believe is the optimal amount of KMC hours in one day?

Do you wait for the baby to wake up before:

Giving medication

Gavage feeding

Counting the respiratory rate

Changing of diaper

Changing position

KMC

Taking blood samples

Have you undergone training in lactation and breastfeeding counselling?

No.

One hour

Two-four hours

One day seminar or more.

How are mothers trained in lactation and feeding of their baby at your ward?

What important steps are taken in your ward to create healing environment for the neonates?

Do you believe that preterm babies feel pain?

Yes

No

When do you perform hand hygiene?

Before patient contact

Before an aseptic task

After body fluid exposure

After patient contact

After contact with patient surroundings

What is the normal temperature range for a newborn baby?

When do you measure the baby's temperature manually?

Supplement S2 Survey
